# Development of a Super-Resolution Scheme for Pediatric Magnetic Resonance Brain Imaging Through Convolutional Neural Networks

**DOI:** 10.3389/fnins.2022.830143

**Published:** 2022-10-25

**Authors:** Juan Manuel Molina-Maza, Adrian Galiana-Bordera, Mar Jimenez, Norberto Malpica, Angel Torrado-Carvajal

**Affiliations:** ^1^Medical Image Analysis and Biometry Lab, Universidad Rey Juan Carlos, Madrid, Spain; ^2^Department of Radiology, Hospital Universitario Quirónsalud, Madrid, Spain

**Keywords:** deep learning (DL), magnetic resonance imaging (MRI), pediatric imaging, sedation, super-resolution (SR), convolutional neural networks (CNN)

## Abstract

Pediatric medical imaging represents a real challenge for physicians, as children who are patients often move during the examination, and it causes the appearance of different artifacts in the images. Thus, it is not possible to obtain good quality images for this target population limiting the possibility of evaluation and diagnosis in certain pathological conditions. Specifically, magnetic resonance imaging (MRI) is a technique that requires long acquisition times and, therefore, demands the use of sedation or general anesthesia to avoid the movement of the patient, which is really damaging in this specific population. Because ALARA (as low as reasonably achievable) principles should be considered for all imaging studies, one of the most important reasons for establishing novel MRI imaging protocols is to avoid the harmful effects of anesthesia/sedation. In this context, ground-breaking concepts and novel technologies, such as artificial intelligence, can help to find a solution to these challenges while helping in the search for underlying disease mechanisms. The use of new MRI protocols and new image acquisition and/or pre-processing techniques can aid in the development of neuroimaging studies for children evaluation, and their translation to pediatric populations. In this paper, a novel super-resolution method based on a convolutional neural network (CNN) in two and three dimensions to automatically increase the resolution of pediatric brain MRI acquired in a reduced time scheme is proposed. Low resolution images have been generated from an original high resolution dataset and used as the input of the CNN, while several scaling factors have been assessed separately. Apart from a healthy dataset, we also tested our model with pathological pediatric MRI, and it successfully recovers the original image quality in both visual and quantitative ways, even for available examples of dysplasia lesions. We hope then to establish the basis for developing an innovative free-sedation protocol in pediatric anatomical MRI acquisition.

## 1. Introduction

Magnetic Resonance Imaging (MRI) is the key modality for obtaining pathophysiological information in a non-invasive manner, while avoiding the use of ionizing radiation that may be harmful to patients, causing them short- or long-term damaging effects. By using MRI, it is possible to obtain high-resolution images describing the anatomy, function, diffusion, etc. of the patient and capture decisive details. Although there are no major issues among most of the adult populations, pediatric MRI acquisition represents, however, an important challenge, as their cooperation becomes more difficult to attain for the clinical trial success. Children need to stand still and immobile during the examination and get to tolerate the claustrophobic environment inside the scanner. However, this is challenging and motion artifacts appear as a direct consequence, frequently worsening the image quality and sharpness, thus, restraining its usability as a diagnosis and treatment tool. In this scenario, and also due to intense noise and the aforementioned restriction of space inside the MRI scanner, sedation is very advisable for examinations of children in most situations, as stopping an MRI scan is expensive and ineffective, hence the failure rate needs to be mitigated (Schulte-Uentrop and Goepfert, 2010). Nevertheless, despite these artifacts can be refrained with the aid of sedation or anesthesia and reach a high image quality, children could suffer potential long-term neurological and cognitive side effects, such as hallucinations or nightmares, and also cardiovascular affectations, like hypertension, bradycardia, alterations in mean arterial pressure, and myocardial depression (Slovis, 2011; Ahmad et al., 2018). Additionally, because as low as reasonably achievable (ALARA) principles are considered for all imaging studies -especially pediatric ones- the main goal is to restrict its dispense as much as possible and limit it only to imperative cases.

In this sense, there exist distraction methods that have revealed a reduction in the percentage of sedated infants compared to control groups (Harned Ii and Strain, 2001; McGee, 2003; Khan et al., 2007), so parents' stress decrease substantially because sedation and its associated recovery time are luckily skipped. One of the most common distraction methods consists of wearing video goggles and earphones in order to watch and listen to films as a distraction during the examination. Another way to calm children and infants during the acquisition is displaying a video on a mobile screen monitor that can be adjusted according to any position of the patient inside the scanner. Moreover, a certified child-life specialist gets children ready to behave correctly in the scanner and attract their attention during the examination with the direct effect of improving image quality. Sleep manipulation might represent an alternative way to handle children's movements (Edwards and Arthurs, 2011). Deprivation of sleep before the examination has been demonstrated to prevent infants from sedation and acts as a substitute for pharmacological sleep induction.

While all these techniques are extremely useful in shortening scan time, they still result insufficient to avoid sedation in some pediatric patients. In this context, ground-breaking concepts and novel technologies can help to find a solution to these challenges while helping in the search for underlying disease mechanisms. Very recently, the feasibility and acceptability of MRI imaging studies without anesthesia have been demonstrated in adolescent populations with moderate or severe neuropathic pain (Verriotis et al., 2020), or autism spectrum disorders (Smith et al., 2019). In this sense, post-MRI-acquisition super-resolution (SR) methods play an essential role. SR methods can provide high resolution (HR) images from low resolution (LR) images and consequently achieve a resultant image quality similar to ideal HR images with a shorter acquisition time.

Traditional mathematical methods can address the SR problem in MRI. For instance, Peled and Yeshurun (2001) and Yan and Lu (2009) use the iterative back projection method in Diffusion Tensor images and anatomical images, respectively. In the case of Gholipour et al. (2010), a stochastic regularization process is applied to brain fetal MRI. Another popular implementations are sparse methods, which subdivide the image and store the result in a dictionary. Lustig et al. (2007) made subimage sampling for sparse coding on the frequency domain, and Zhang et al. (2019) succeeded to recover details from neonatal T1 and T2-weighted MRI, with the help of older children's images. However, all these classical methods for SR come with performance limitations when applied to pediatric MRI. With that in mind, it is preferred to develop a scheme that profits from deep learning (DL) tools to generate the SR image, as they imply an improvement compared with results using classical methods.

We can find convolutional neural network (CNN) implementations in the literature that successfully solve the SR problem using MRI. Pham et al. (2019) and Chaudhari et al. (2018) implemented a 3D CNN with no feature dimension reduction and evaluated the network for many scaling factors. They also demonstrate that one single network gets to deal with more than one scaling factor. In the case of Pham et al. (2019), the same number of filters is applied for each layer and they use residual operations and a single skip connection. Qualitative evaluation is carried out from HR neonatal reconstructions as the ground truth of real LR data is not available. In Zeng et al. (2020), a fully dense connected cascade network is developed and they compared the results with a Unet, which is the base architecture in our work. Chen et al. (2018) also proposed a 3D densely connected network for brain MRI SR with the same number of filters inside the dense block. Pham et al. (2017) designed a 3D CNN with variation in the number of filters depending on the layer, while Du et al. (2020) opt for a residual CNN whose input is individual 2D MRI slices. As well, Qiu et al. (2020) combine a three hidden layer 2D CNN with a sub-pixel convolutional layer using knee MRI and Zeng et al. (2018) carry out a sophisticated approach where they benefit from distinct modality information and the architecture implies two consecutive CNNs.

Apart from CNN development in brain MRI SR, some works based on Generative Adversarial Networks (GANs) have been published in the last few years. Sánchez and Vilaplana (2018) used T1 MRI for two different scaling factors, and Lyu et al. (2018) takes as input T2 individual MRI slices for training two kinds of GANs. Wang et al. (2020) implement a 3D GAN framework with T1 images. Finally, You et al. (2020) and Tan et al. (2020) make a more elaborated implementation of the GAN framework.

In this work, we exploit recent development in Graphics Processing Units (GPU) and we present a DL approach to address the SR problem for pediatric MRI. Our CNN is based on the form of a Unet autoencoder and we get to successfully recover the resolution from pediatric LR MRI. The CNN architecture includes skip connections, residual operations, and feature maps with distinct dimensions depending on the layer, allowing the network to learn features with many levels of complexity. Using consecutive convolutions, we intend to extract critical features that determine the SR process during the encoding stage. Then, the objective is to reconstruct the output from deep features maps as dimension and domain from input and output MRI of CNN need to be the same in order to compare both at the evaluation stage.

## 2. Materials and Methods

### 2.1. Dataset

An open dataset containing brain MRI volumes of 155 healthy controls has been used for the main implementation and technical assessment of our DL architecture (Richardson et al., 2018). This dataset is composed of 33 adults (20 female; 24.8 ± 5.3 years, range of 18–39 years) and 122 children (64 female; 6.7 ± 2.3 years, range of 4–12 years). Anatomical T1-weighted and functional MRI volumes have been acquired on a Siemens TIM Trio 3T MRI scanner (Siemens Healthineers, Erlangen, Germany) located at the *Athinoula A. Martinos Center for Biomedical Imaging (Boston, MA, USA)*. Anatomical volumes were obtained with a 32 channel head coil using an MPRAGE sequence (*TR* = 2.53 s, *TE* = 1.64 ms, *FlipAngle* = 7°, 1 mm isotropic voxel) including two different matrix sizes: 176 × 256 × 256 and 176 × 192 × 192. Specific head coils have been used for subjects younger than 5 years.

An additional dataset composed of 12 children (3 female, 9.29 ± 3.76; range of 3–16 years) brain MRI volumes diagnosed with different types of dysplasia and presenting different lesions has been used for a more detailed clinical assessment (Table 1). Anatomical T1-weighted volumes have been acquired on two different General Electric (GE) 3T MRI scanners, a Signa HDxt 3.0T and a Signa Premier(GE Healthcare, Waukesha, WI, USA) located at *Quirónsalud Madrid University Hospital (Pozuelo de Alarcón, Madrid)*. Among them, 8 volumes were obtained with an 8 channel head coil using an EFGRE3D sequence (*TR* = 9.244 ms, *TE* = 3.428 ms, *TI* = 750 ms, *FlipAngle* = 10°, 1.13mm isotropic voxel) and 4 of them were acquired with a 48 channel head coil using an MPRAGE sequence (*TR* = 2.2 s, *TE* = 2.8 ms, *FlipAngle* = 8°, 1 mm isotropic voxel). The use of these images for the present study was approved by the local Institutional Review Board.

**Table 1:** Demographic data of the pediatric dataset used for the clinical evaluation of the algorithm.

**ID#**	**Age**	**Sex**	**Disease**	**MRI sequence (Scanner)**
001	8	F	Simple cortical dysplasia	EFGRE3D (Signa HDxt)
002	9	F	Taylor dysplasia	EFGRE3D (Signa HDxt)
003	11	M	Bilateral opercular syndrome	EFGRE3D (Signa HDxt)
004	16	M	Aqueductal stenosis; Opercular syndrome; Heterotopia; Cystic brain lesion	EFGRE3D (Signa HDxt)
005	6	F	Opercular syndrome	EFGRE3D (Signa HDxt)
006	8	M	Taylor dysplasia	EFGRE3D (Signa HDxt)
007	5	M	Cingulum dysplasia	EFGRE3D (Signa HDxt)
008	13	M	Opercular syndrome	EFGRE3D (Signa HDxt)
009	3	M	Opercular syndrome; Cerebral fissure malformation	MPRAGE (SIGNA Premier)
010	8	M	Cortical dysplasia	MPRAGE (SIGNA Premier)
011	13	M	Taylor dysplasia	MPRAGE (SIGNA Premier)
012	6	M	Cortical dysplasia	MPRAGE (SIGNA Premier)

### 2.2. Data Preprocessing

#### 2.2.1. Quality Assurance and Size Standardization

Quality assurance (QA) in the open database revealed that from the initial 155 healthy controls, 32 children volumes (matrix size 176 x 192 x 192) demonstrated evident imaging artifacts that could negatively impact our procedure and, as a consequence, they were excluded from the final dataset. Thus, 123 healthy controls were selected after QA: 33 adults (20 female; 24.8 ± 5.3 years, range of 18–39 years) and 90 children (46 female; 7.1 ± 2.0 years, range of 4–12 years).

The final matrix size of the selected images after QA is 176x256x256. Zero-padding was applied on both sides in the first dimension to ease later patching of the volumes when fed to our CNN architecture while maintaining the brain centered in the final volume. Additionally, the background was homogenized using a binary mask. The images were not denoised, bias corrected, or registered as part of the pre-processing stage.

The same procedure was applied to the clinical pediatric dataset. No images were discarded in this process. However, as our original pipeline was developed for a matrix size of 256 × 256 × 256 in the open access dataset, we resized all MRI volumes from 512 × 512 × 512 to 256 × 256 × 256.

#### 2.2.2. Creation of Low Resolution Database

The selected databases are composed of HR images. Therefore, the first step is the artificial production of LR images from HR original ones. First, we simultaneously apply both downsampling and smoothing operations convolving a 3D cubic gaussian kernel of 5 voxels size (σ = 1) with the MRI volume, in one of each N voxels per dimension. If the original volume dimensions are (*d*_1_, *d*_2_, *d*_3_), dimensions of HR image downsampled will be (*d*_1_/*N, d*_2_/*N, d*_3_/*N*) with N as the selected scaling factor. This way of deriving the LR counterparts is in consonance with previous works in the state of the art (Rueda et al., 2013; Shi et al., 2015; Pham et al., 2017, 2019; Zeng et al., 2018).

Low resolution downsampled images are then interpolated to HR image size. Although interpolation is an image transformation that could add noise to the downsampled image, it avoids fitting the CNN to an specific scaling factor. In this manner, the designed CNN does not depend on LR image size, and it becomes a robust method to implement on any image size. Hence, LR images are rescaled to original HR image size (*d*_1_, *d*_2_, *d*_3_). New intensity values are created among existing ones to get the necessary matrix size. In the majority of the mentioned studies that have applied CNNs to SR of MRI, bicubic splines is the key method for interpolation, thus used in this work.

Then, we apply min-max normalization to all MRI volumes. The intensity values of every image are transformed to a common [0, 1] range. As every LR volume is created from an HR image, each LR normalization takes the arguments (minimum and maximum intensity values) from the associated HR image.

#### 2.2.3. Volumes Patching

Convolutional neural network architecture determines the way images feed the network. This work explores two network modalities: 2D and 3D. 3D CNNs receive 3D volumes as input, but not full LR volumes in our case. NVIDIA graphic cards cannot store all parameters for the whole volume because of their limited memory, which forces us to extract patches from the images. Patches are cubic volumes of size 32 and they are extracted in train and validation sets using a stride equal to 32, with no overlap, in order to be differentiated from test patching. This patching process is carried out identically for both input and label images of the network. On the other hand, 2D CNNs receive two-dimensional inputs which are complete individual slices of size 256 x 256.

The data augmentation process is carried out to assure a sufficient amount of information and get CNN generalization. It consists of randomly rotating each volume in every dimension by selecting a random angle within the interval [−30, 30] degrees. We select this interval range of angles as 30 degrees is the maximum possible shift of the subject position inside the MRI scanner.

### 2.3. Hardware and Software

Computational equipment includes a processor *Intel(R) Core(TM) i9-10980XE CPU, 128 GB RAM memory, Ubuntu 20.04 operating system*, and *1 TB hard disk storage*. Data preprocessing has been developed using *Python 3*, and CNNs were designed and trained with *Tensorflow 2* library using *GPU NVIDIA GeForce RTX 3080*.

### 2.4. Super-Resolution Formulation

The DL-based SR process aims to estimate an HR image $\hat{\text{X}}$ from an LR image **Y**∈ℝ^*n*^ that is simulated from an HR ground-truth image **X**∈ℝ^*m*^. Thus, the connection between **X** and **Y** is formulated as follows:


(1) $$\begin{array}{l} \text{Y}=\text{T}\left(\text{X}\right)=\left({\text{D}}_{↓}\text{S}\right)\text{X}+\text{N} \end{array}$$

with **T**∈ℝ^*m, n*^ (*m*>*n*) as the general transformation, in our case, based on applying to the ground truth HR image a combination of downsampling **D**_↓_ and smoothing **S** processes. **N** indicates the noise related to the transformation. For the purpose of finding the inverse mapping estimation, it is convenient to minimize a least-squares cost function:


(2) $$\begin{array}{l} \hat{\text{X}}=\underset{X}{\text{argmin}}\;||\text{X}-{\text{T}}^{-1}\left(\text{Y}\right)|{|}^{2}=\underset{X}{\text{argmin}}\;||\text{X}-\text{R}\left({\text{I}}^{↑}\left(\text{Y}\right)\right)|{|}^{2} \end{array}$$

being **T**^−1^ an ensemble of a recovery matrix **R**∈ℝ^*m, m*^ and an upsampling operator **I**^↑^ regarding the spline interpolation of LR image **Y**. Since we have multiple subjects available, $\hat{\text{R}}$ can be achieved by minimizing the following additive function:


(3) $$\begin{array}{l} \hat{\text{R}}=\underset{R}{\text{argmin}}\;\overset{N}{\underset{i=1}{\sum }}||{\text{X}}_{i}-\text{R}\left({\text{I}}^{↑}\left({\text{Y}}_{i}\right)\right)|{|}^{2} \end{array}$$

where N is the number of subjects available for generating the model. Finally, the optimal $\hat{\text{R}}$ learnt allows to produce an SR estimation $\hat{\text{X}}$ from a new LR image **Y**:


(4) $$\begin{array}{l} \hat{\text{X}}=\hat{\text{R}}\left({\text{I}}^{↑}\left(\text{Y}\right)\right) \end{array}$$

### 2.5. CNN Architecture

The neural network chosen to perform SR is the Unet autoencoder, whose basic structure and function are presented in Ronneberger et al. (2015). This structure is taken as the core basis to insert residual operations to improve performance. In the encoding stage, several downsampling transformations are performed by max pooling operations (pool value of 2 voxels per dimension), and the number of filters is double increased. Moreover, the decoding stage contains transposed convolutions (stride value of 2 voxels per dimension) as upsampling operations, where the number of filters is consecutively halved to achieve the initial image size. In the course of the decoding stage, after each transposed convolution, skip connections concatenate feature maps with their homologous encoding phase to preserve information from multiple scales and complexities. Apart from the dimensions of convolution kernels, 2D and 3D CNNs have identical architecture.

Our proposed architecture is divided into 9 stages. The first four stages are focused on encoding, the fifth one is the latent space, whereas the last four ones belong to the decoding stage. The first two stages in encoding and last two stages on decoding have two convolutional blocks each, whereas the rest of the stages contain three convolutional blocks, and we get 23 convolution blocks in all. Each of those blocks consists of a convolutional layer followed by batch normalization and *ReLU* activation function. Also, we have one final convolution at the end of the network and 4 transposed convolutions in the decoding stage, which makes a total of 28 convolutional layers for the whole CNN.

All convolutional layers use (3,3,3) kernel size except the last one. We take into account that the model accuracy may worsen as the network depth increases so the number of convolutional layers is the minimum needed to succeed with generalization. Also, we tested the model with dilated convolutions but they have been discarded as they did not improve the performance. This demonstrates that a more complex and sophisticated implementation of neural networks does not always give better results.

**Figure 1:**
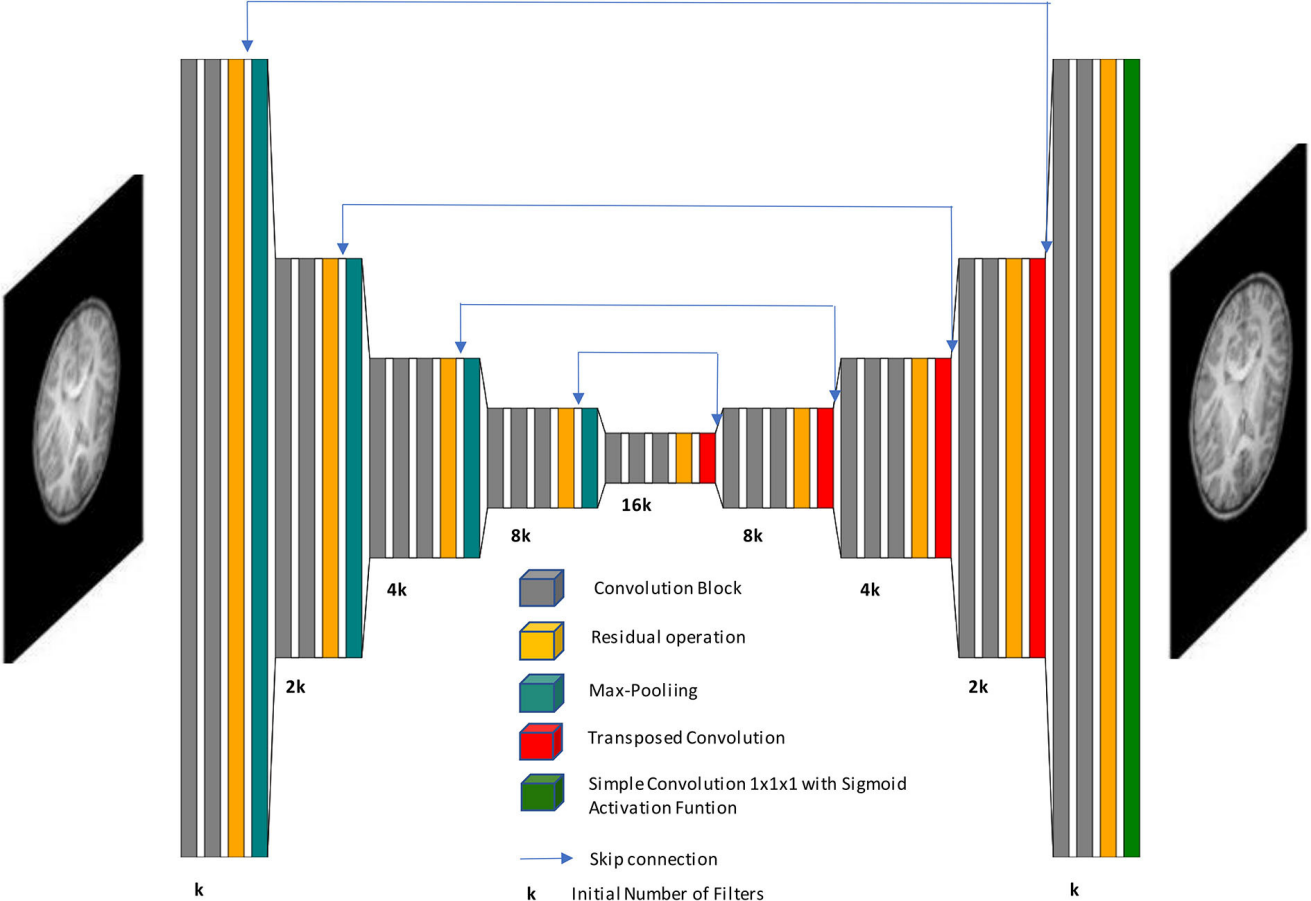
Schematic representation of our network architecture. Our autoencoder implementation processes low-resolution images to generate high-resolution images.

Residual operations intend to avoid the gradient vanishing problem and are located before each max pooling layer and after each transposed convolution. They append the input features maps to the output feature maps of the current stage. These input maps are previously convolved with a kernel size (1,1,1). Finally, the last convolutional layer employs (1,1,1) kernel size and a sigmoid activation function to bound the intensity values, in case some exceeds the interval [0, 1]. Figure 1 shows a general overview of the CNN architecture.

### 2.6. Experimentation

Once data preprocessing is implemented on the original volumes and the model CNN architecture is defined, subjects are divided into different subsets. The main purpose of this work is to significantly improve the resolution of MRI among the infant population. As mentioned before, there are images of adults in the healthy database, and we benefit from them to start the training process and provide a first learning step for our model.

Although adult subjects are not sufficient to reach the expected performance, they generate a first model approach as a starting point for training with children's data. By doing so, the variability and size of the database are increased and we gain a better model generalization. In this manner, we apply transfer learning by retraining healthy children from best adults model, which allows to attain faster and closer the optimal solution of the SR proposal.

There are 33 adult volumes available and they are assigned to the training set (80%, 26 volumes) and test set (20%, 7 volumes). The reason why there is no validation group in adults is that these subjects help to get a first approach to the model but not to evaluate the final model performance, as this work is focused on children SR. For 90 total healthy children, test and validation sets correspond to 18 volumes each (20%) and the training set contains 54 volumes (60%). In the case of dysplasic patients, we obtained worse results doing transfer learning from the healthy children model than when processing these volumes directly with the aforementioned model. For that reason, this last set of subjects is not divided into subsets and they just form together with the test set (12 volumes).

As optimizer, we chose Adam with an initial learning rate of 0.0001, which is reduced *e*^0.2^ times if validation loss stops decreasing for more than 4 epochs, being 0.00001 its minimum possible value. Our loss function is the sum of the mean absolute error and a gradient absolute error function. This allows to give value to regions, such as borders or contours, where information could be easily missed otherwise while focusing also on the rest of the image.

Adult and children models have been trained for 50 and 100 epochs, respectively. Those number of epochs are large enough to achieve the convergence of the model. The model selected to perform an evaluation on the test set is the one that gets a smaller value of loss function on the validation group, to avoid both underfitting and overfitting. Furthermore, the loss function is calculated in every iteration only on tissue voxels to prevent background noise from influencing the learning process. Also, batch size has been defined as 12 patches for 3D CNN and 8 patches for 2D CNN, which means that backpropagation updates the parameters after that number of patches. The number of initial filters is 64 for both 3D and 2D CNNs. Even though 2D kernels imply less memory to store, the input patch in 2D CNN contains more voxels than its 3D counterpart and we can not manage with a higher number of filters. Kernel weights are initialized randomly.

Different experiments are performed for every CNN designed. Each CNN (2D and 3D) is trained with three distinct scaling factors (*x2, x3, x4*) separately. The goal is to analyze at which point there is the best trade-off between the quality of HR estimated image and a significant reduction of MRI acquisition time. In a summary, 6 different training experiments are developed. Spline interpolation is not considered an experiment itself but it is however included in later results for comparing it with CNN-based methods.

### 2.7. Evaluation and Metrics

Before proceeding with the evaluation stage, the complete estimated volume must be reconstructed by merging the patches or slices generated by the CNN. Image reconstruction for 2D network output is straightforward as it simply consists of attaching one slice above the other. 3D reconstruction requires a more sophisticated procedure. If we merely allocated the produced patches to recover the total volume, borders among them would be evident when visualizing the image. Thus, we extract patches from interpolated LR test images with a stride value lower than the patch side size (in our case this size is 32 voxels and stride value is defined as half size, that is, 16 voxels). Once the CNN outputs the SR estimated patches, they are placed on the final volume and we extract the inner cube of 16 side voxels.

The output of our CNN model is not a single scalar value but a complete image. Thus, we measure the model performance with those mathematical functions able to compare the similarity between the output image and the ground truth image. Three metrics are used in this stage: Peak Signal-to-Noise Ratio (PSNR), Structural Similarity Index Metric (SSIM), and Mean Absolute Error (MAE).

Peak Signal-to-Noise Ratio tries to estimate how noise affects image quality. Its value is commonly expressed in decibel (dB) logarithmic scale:


(5) $$\begin{array}{l} PSNR\left(\hat{\text{y}},\text{y}\right)=20\log_{10}\left(\frac{MAX}{\sqrt{MSE\left(\hat{\text{y}},\text{y}\right)}}\right)\;\left[dB\right] \end{array}$$

where *MAX* represents the maximum intensity value of the HR ground truth image, that is 1 in our case, and *MSE* refers to Mean Squared Error.

Mean Absolute Error measures the mean magnitude of errors between images. It calculates the average absolute differences between each pair of equivalent voxels, and it is expressed in intensity values of the image:


(6) $$\begin{array}{l} MAE\left(\hat{\text{y}},\text{y}\right)=\frac{1}{N}\overset{N}{\underset{i=1}{\sum }}|\hat{\text{y}}\left(i\right)-\text{y}\left(i\right)| \end{array}$$

Structural similarity index metric is a metric that quantifies the degradation of the estimated image by extracting 3 key features of the images: luminance, contrast, and structure. It is a non-dimensional metric, much more coherent with human visual perception and is able to imitate it in a quantitative manner. The range of possible values fluctuates in the interval [−1, +1], with 1 meaning exact images. Mathematically, it can be expressed as follows:


(7) $$\begin{array}{l} SSIM\left(\hat{\text{y}},\text{y}\right)=\frac{\left(2\mu_{\hat{\text{y}}}\mu_{\text{y}}+c_{1}\right)\left(2\sigma_{\hat{\text{y}}\text{y}}+c_{2}\right)}{\left(\mu_{\hat{\text{y}}}^{2}+\mu_{\text{y}}^{2}+c_{1}\right)\left(\sigma_{\hat{\text{y}}}^{2}+\sigma_{\text{y}}^{2}+c_{1}\right)} \end{array}$$

where *c*_1_ and *c*_2_ are regularization constants of the metric itself, $\sigma_{\hat{\text{y}}\text{y}}$ is the images joint covariance, $\mu_{\hat{\text{y}}}$ and μ_**y**_ are the averages of images, and $\sigma_{\hat{\text{y}}}^{2}$ y $\sigma_{\text{y}}^{2}$ are the images variances.

Although it makes sense to calculate metrics only on the brain tissue using the binary mask and preventing the background of biasing and distorting the evaluation, most of the related literature provides the results on the whole volume and we, therefore, provide metrics for the complete image. While PSNR and SSIM are relative measures and do not depend on the intensity values scale, MAE is however sensitive to it. Thus, we indicate the MAE metric in terms of [0, 255] standard range of possible values.

### 2.8. Statistical Analysis

Statistical analysis was performed using Python. The quantitative performance of the different methods was assessed by comparing the metrics computed between ground truth images and super-resolved images, using a repeated measures analysis of the variance (ANOVA) to evaluate the effect of the method (interpolation, 2D CNN, 3D CNN), scaling factor (x2, x3, x4), and their interaction, followed by paired-samples Wilcoxon signed rank tests to assess if the performance of each CNN was significantly different from the rest of the methods. Statistical significance was considered when the *p*-value was lower than 0.05.

For the purpose of evaluating the detection of dysplasia lesions in our SR images, an expert radiologist performed two different tests. The first one consists of providing a binary answer whether the lesion can be diagnosed for each of the patients. We only have positive cases in our second database so the sensitivity calculated provides a measure that represents the percentage from all affected SR images where the physician is able to still detect the dysplasias. The second test performed is a 5-point Likert scale. It assigns to each image one of five different levels, where level one means it is impossible to detect the lesions and level five indicates the dysplasia is found perfectly and easily.

## 3. Results

In this section, we provide both qualitative and quantitative assessments of our methods. These two assessments were also used to infer which architecture (2D vs. 3D) fits better with the SR task. All models were assessed on pediatric images, and all assessments were performed for the different scaling factors to evaluate the ability of our SR scheme to recover data. Finally, we provide some clinical insights into the best model as assessed in the clinical dataset and a further evaluation of the resulting images in a diagnostic scenario.

### 3.1. Qualitative Assessment

**Figure 2:**
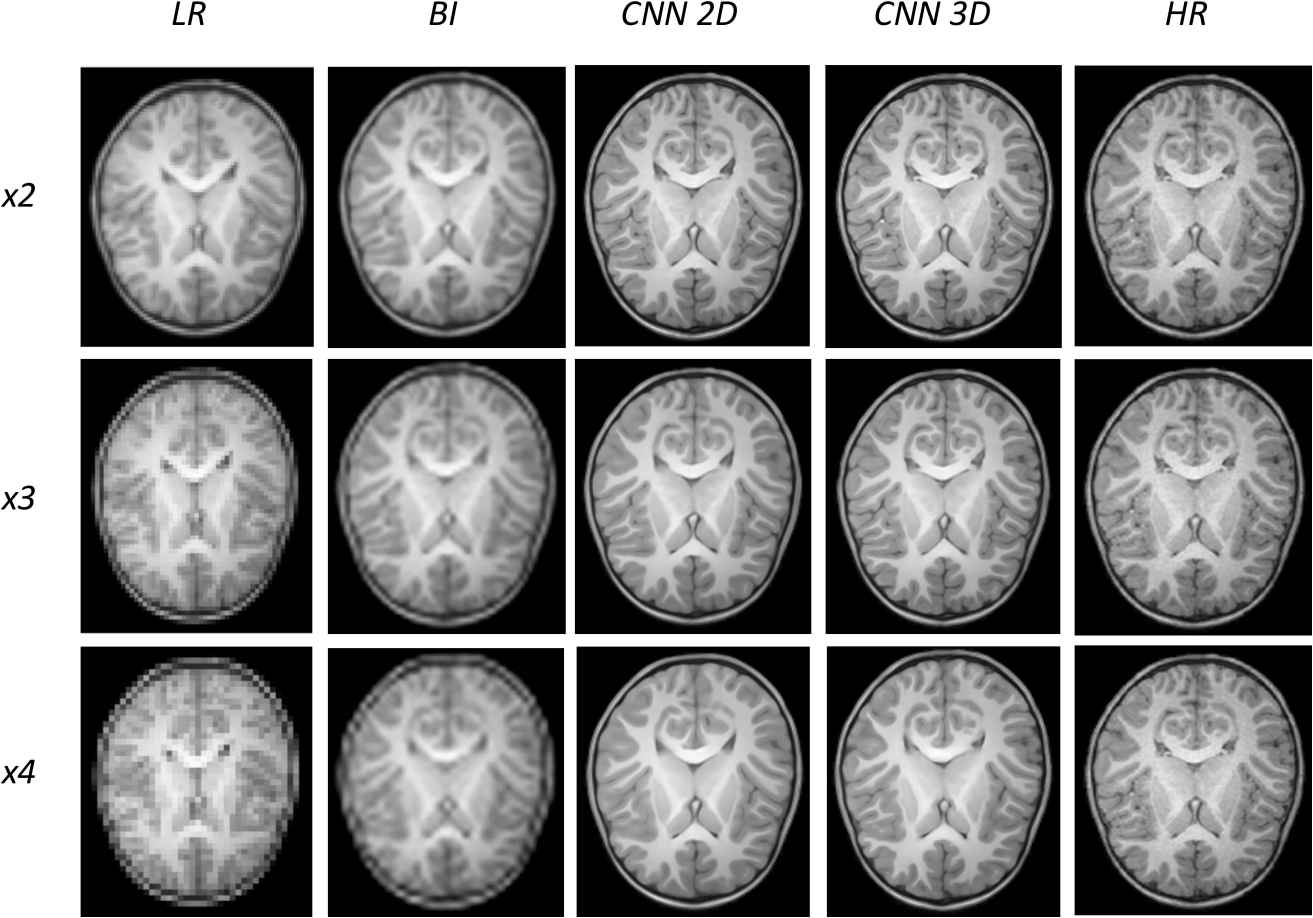
Results of both CNN methods on three scaling factors of super-resolution. A representative slice is shown, together with the original high-resolution image, the low resolution image and the results of bicubic interpolation.

**Figure 3:**
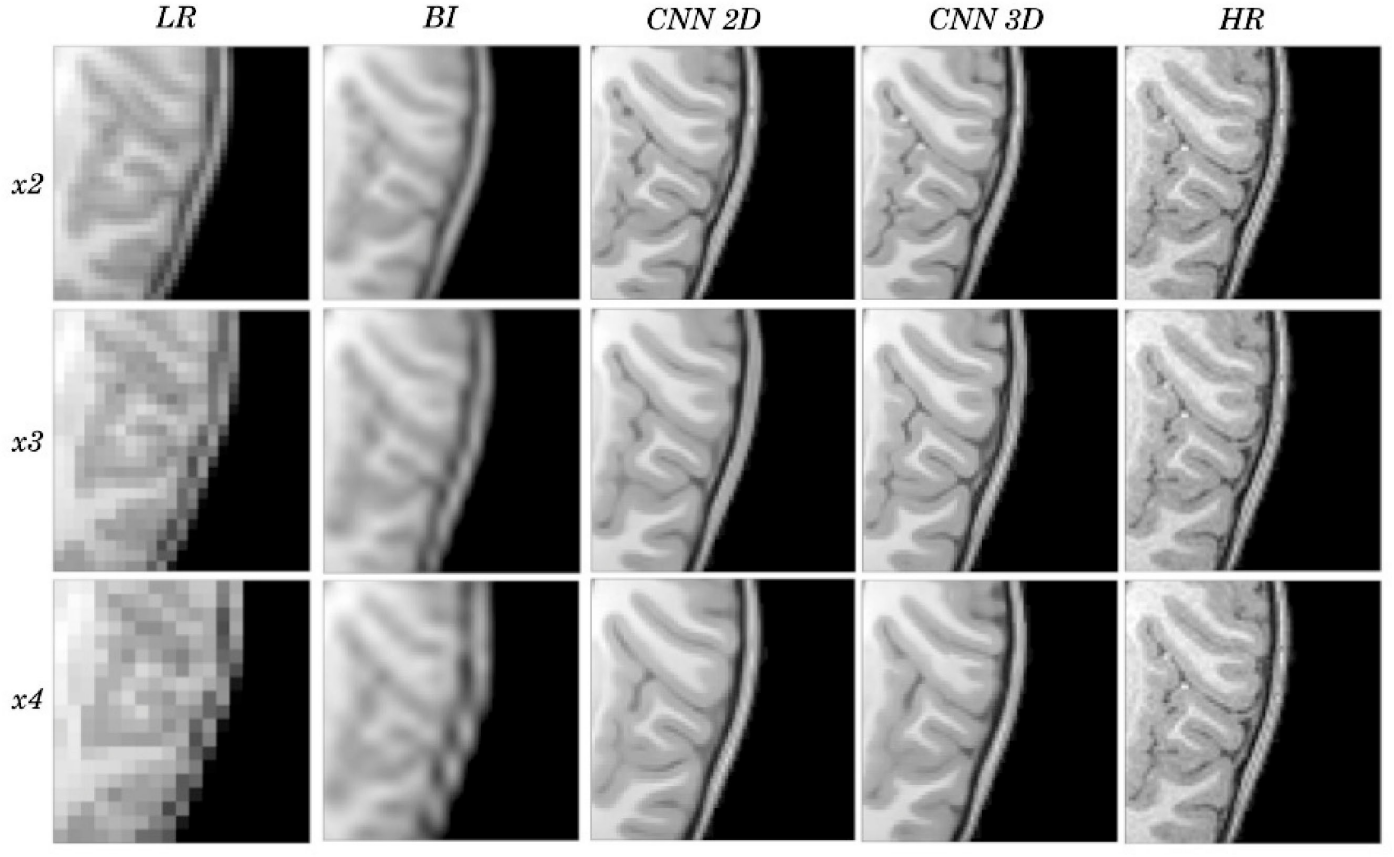
Detail on the images of Figure 2.

An expert radiologist performed a qualitative assessment by visually inspecting the resulting images to confirm the accuracy of our method; our pipeline performed equally well in all cases considered in the original dataset. Figures 2, 3 show a visual comparison between different super-resolution methods (Interpolation, CNN-2D, CNN-3D) and scaling factors (*x2, x3, x4*) compared to the LR input and the HR ground truth image in a representative case of a 5-year-old subject in the dataset. We can see how the bicubic interpolation method provides an overall improvement over LR images. However, despite solving the loss of spatial resolution, the resultant images are still excessively smoothed, presenting a lack of detail and contrast. The detailed definition of structures and contours is not well recovered in the interpolated image because it is based on “blind” learning, just on neighboring information from the LR image, thus the newly generated intensity values are basically noise added to the LR image.

Visual inspection of the images generated by both CNNs (2D and 3D) shows that this problem disappears, and the contours are successfully recovered. The granulation that exists in the original HR image is lost, and our resulting images show smoother intensities than the original ones due to some inherent filtering/denoising performed by the CNN, but the shape of the brain was reconstructed generally well despite patient-specific anatomic variations. Actually, the comparison between the patient-specific HR volume and different SR volumes shows that our method accurately estimates the ground truth, delimiting the contours and differentiating tissues for all scaling factors. Visual inspection of the results show the high quality of the SR estimation and the robustness of the method, which is able to capture details of the different tissues in non smooth areas such as the brain circumvolutions. However, as expected, results from higher scaling factors (*x3* and *x4*) present fewer details than those from *x2* scaling factor, losing certain high pixel intensities. Additionally, the 2D CNN slightly loses contour definition compared to the 3D CNN.

### 3.2. Quantitative Assessment

**Figure 4:**
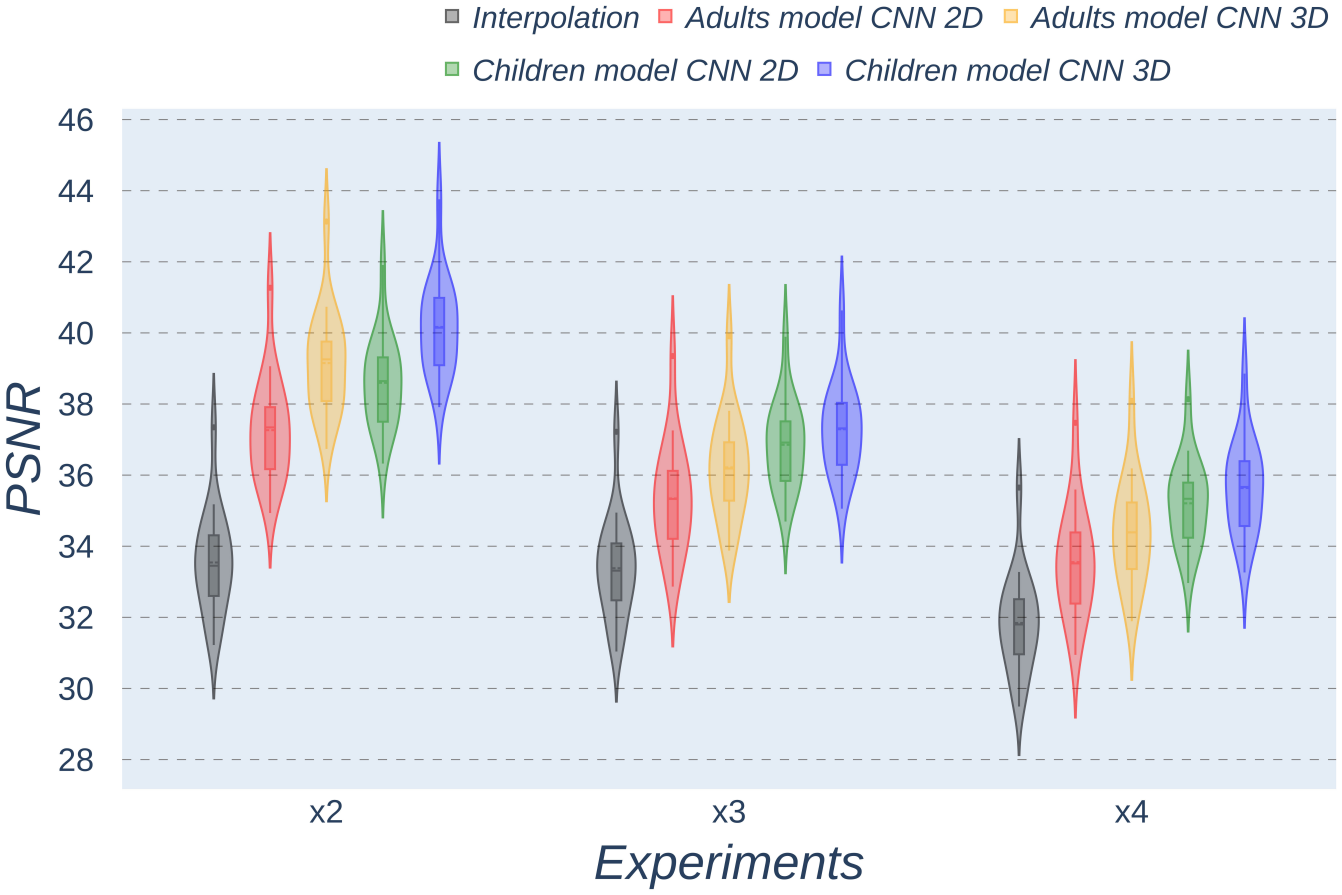
Violin plots for PSNR metric comparing the different methods at the distinct scaling factors. Each violin plot corresponds to one specific experiment, and it is calculated from healthy subjects.

**Figure 5:**
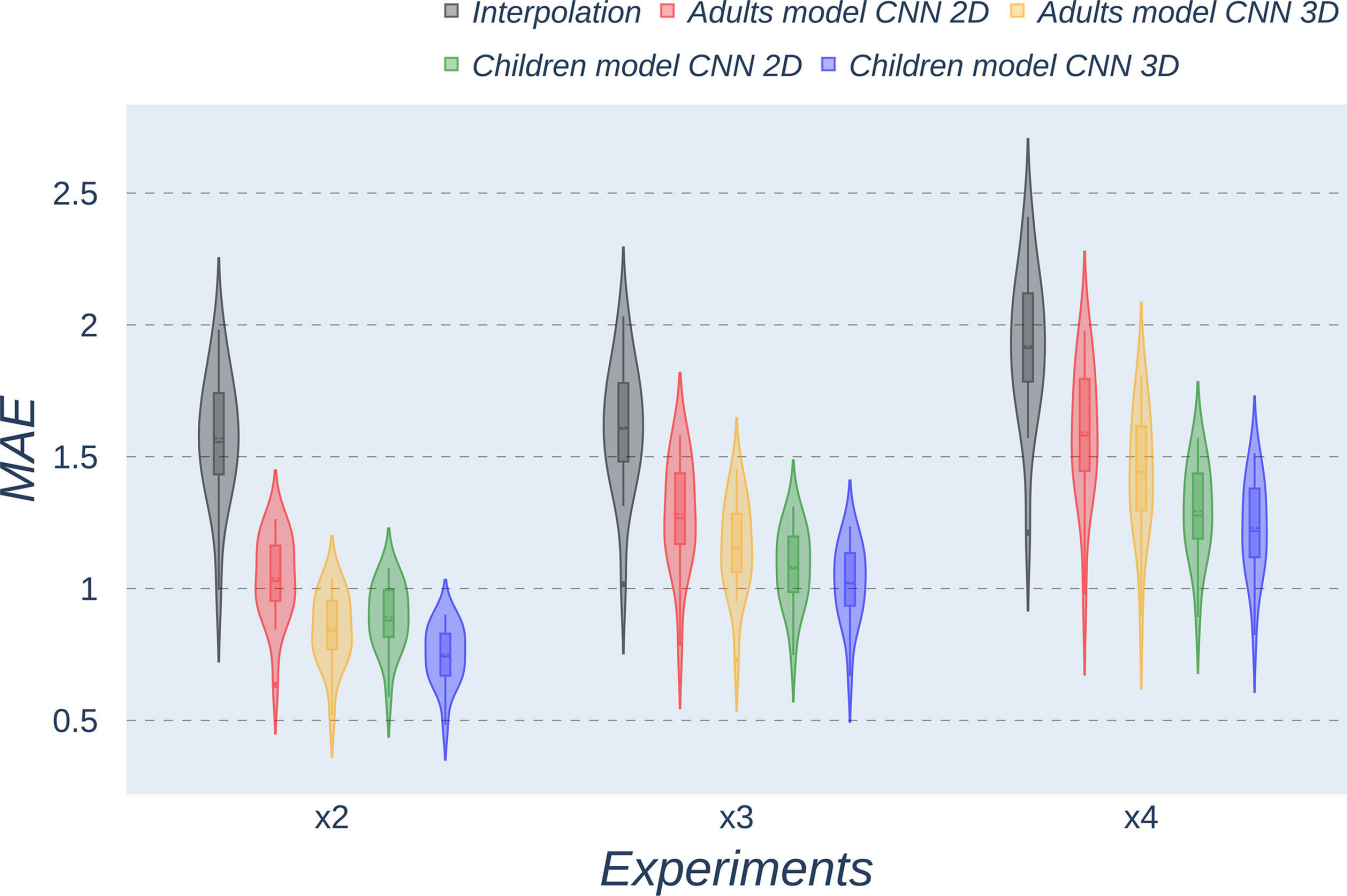
Violin plots for MAE metric comparing the different methods at the distinct scaling factors. Each violin plot corresponds to one specific experiment, and it is calculated from healthy subjects.

**Figure 6:**
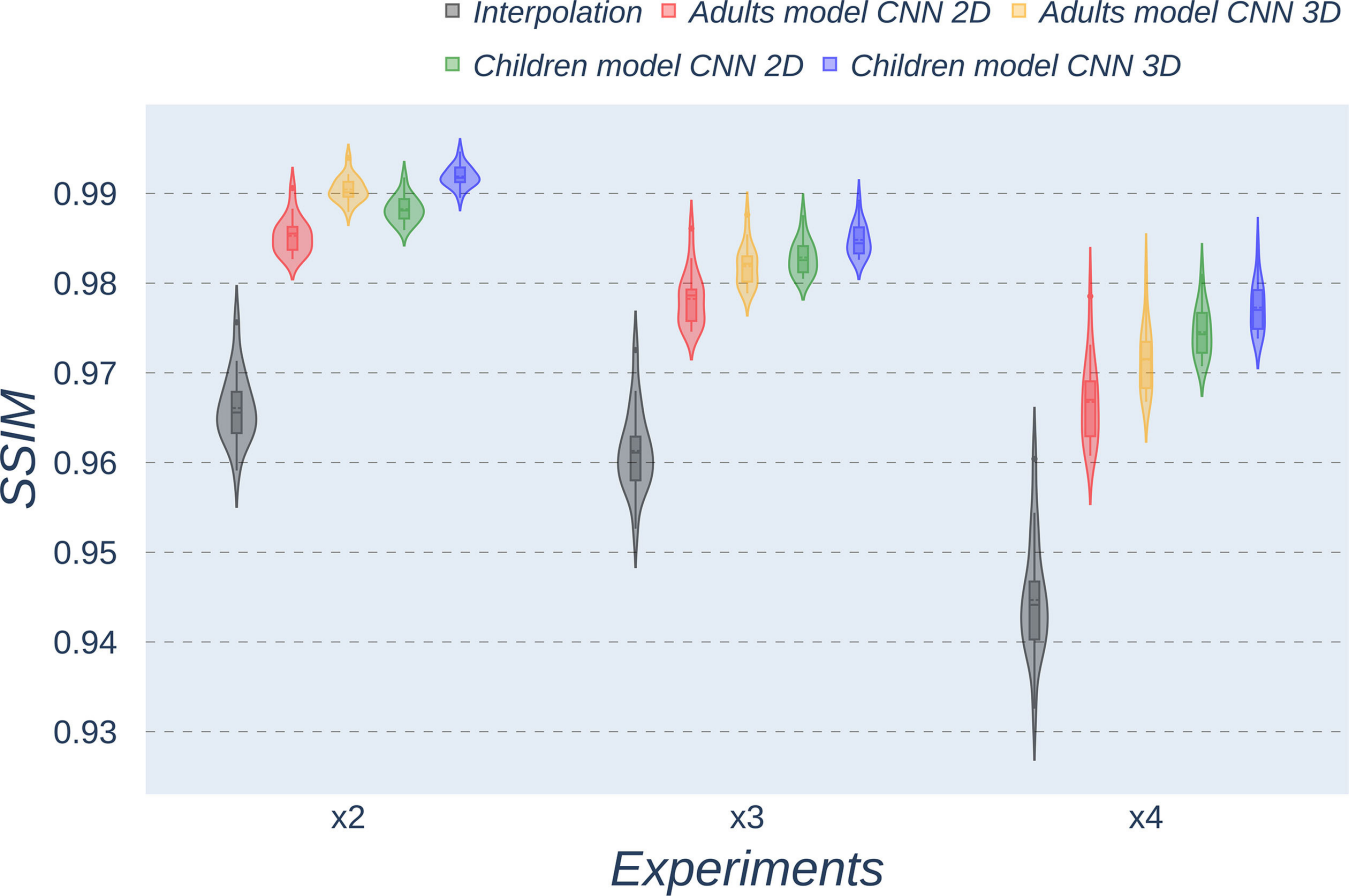
Violin plots for SSIM metric comparing the different methods at the distinct scaling factors. Each violin plot corresponds to one specific experiment, and it is calculated from healthy subjects.

Besides qualitative evaluation, various metrics (PSNR, MAE, and SSIM) have been calculated using the test children's images from the open dataset. Tables 2–4 show the resultant mean and SD for the different metrics, scaling factors, and SR method, computed as the average among children in the dataset. Similarly, Figures 4–6 display the statistical distribution of these participants regarding the same aspects. Adult models are included in the violin plots to show that results improve when performing transfer learning from the adult dataset to the pediatric dataset in all cases considered. Statistical tests are performed to compare interpolation and the pediatric CNNs, not including the adult CNNs in these analyses.

**Table 2:** Results for different metrics and scaling factors among healthy children test set using bicubic interpolation method.

**Healthy children**	**BICUBIC INTERPOLATION**		
	**x2**	**x3**	**x4**
PSNR (dB)	33.543 ± 1.404	33.382 ± 1.400	31.841 ± 1.374
MAE	1.569 ± 0.233	1.610 ± 0.238	1.919 ± 0.280
SSIM	0.966 ± 0.004	0.961 ± 0.005	0.945 ± 0.006

**Table 3:** Results for different metrics and scaling factors among healthy children test set using 2D CNN method.

**Healthy children**	**CNN 2D**		
	**x2**	**x3**	**x4**
PSNR (dB)	38.596 ± 1.239	36.852 ± 1.189	35.207 ± 1.182
MAE	0.892 ± 0.123	1.081 ± 0.144	1.293 ± 0.173
SSIM	0.988 ± 0.001	0.983 ± 0.002	0.974 ± 0.003

**Table 4:** Results for different metrics and scaling factors among healthy children test set using 3D CNN method.

**Healthy children**	**CNN 3D**		
	**x2**	**x3**	**x4**
PSNR (dB)	40.167 ± 1.298	37.290 ± 1.239	35.637 ± 1.273
MAE	0.750 ± 0.107	1.022 ± 0.142	1.229 ± 0.176
SSIM	0.992 ± 0.001	0.985 ± 0.002	0.977 ± 0.003

All metrics reveal a considerable improvement of CNNs compared to the interpolation approach. In particular, the 3D CNN reaches better results compared to its 2D counterpart. This could be mainly due to a leakage of information along the third dimension when using 2D convolutions, affecting recovering continuity of anatomical structures within that dimension. Nevertheless, the 2D version of the CNN represents a valuable alternative in cases where complete volumes cannot be acquired and isolated two-dimensional slices are the only data available.

Analysis of variance test for PSNR reveals a statistically significant effect for the “*method”* in all the scaling factors: *x2, x3*, and *x4* [*F*_(2, 51)_ = 44.91; *p* = 5.65 x 10^−12^], and the SR procedures: interpolation, 2D, and 3D [*F*_(2, 51)_ = 42.71; *p* = 7.73 x 10^−3^]. ANOVA test for MAE also reveals a statistically significant effect for the “*method”* in all the scaling factors: *x2, x3*, and *x4* [*F*_(2, 51)_ = 53.01; *p* = 3.52 x 10^−13^] and the SR procedures: interpolation, 2D, and 3D [*F*_(2, 51)_ = 9.82; *p* = 2.47 x 10^−4^]. In the SSIM case, ANOVA test reveals a statistically significant effect for the “*method”* in all the scaling factors: *x2, x3*, and *x4* [*F*_(2, 51)_ = 307.37; *p* = 3.54 x 10^−29^] and the SR procedures: interpolation, 2D, and 3D [*F*_(2, 51)_ = 87.64; *p* = 3.16 x 10^−17^]. For all three metrics, the decomposition of such statistical results using paired comparisons by means of the Wilcoxon test reveals that all pediatric methods are statistically different from each other at all scaling factors and procedures (*p*′*s* < 0.01).

**Figure 7:**
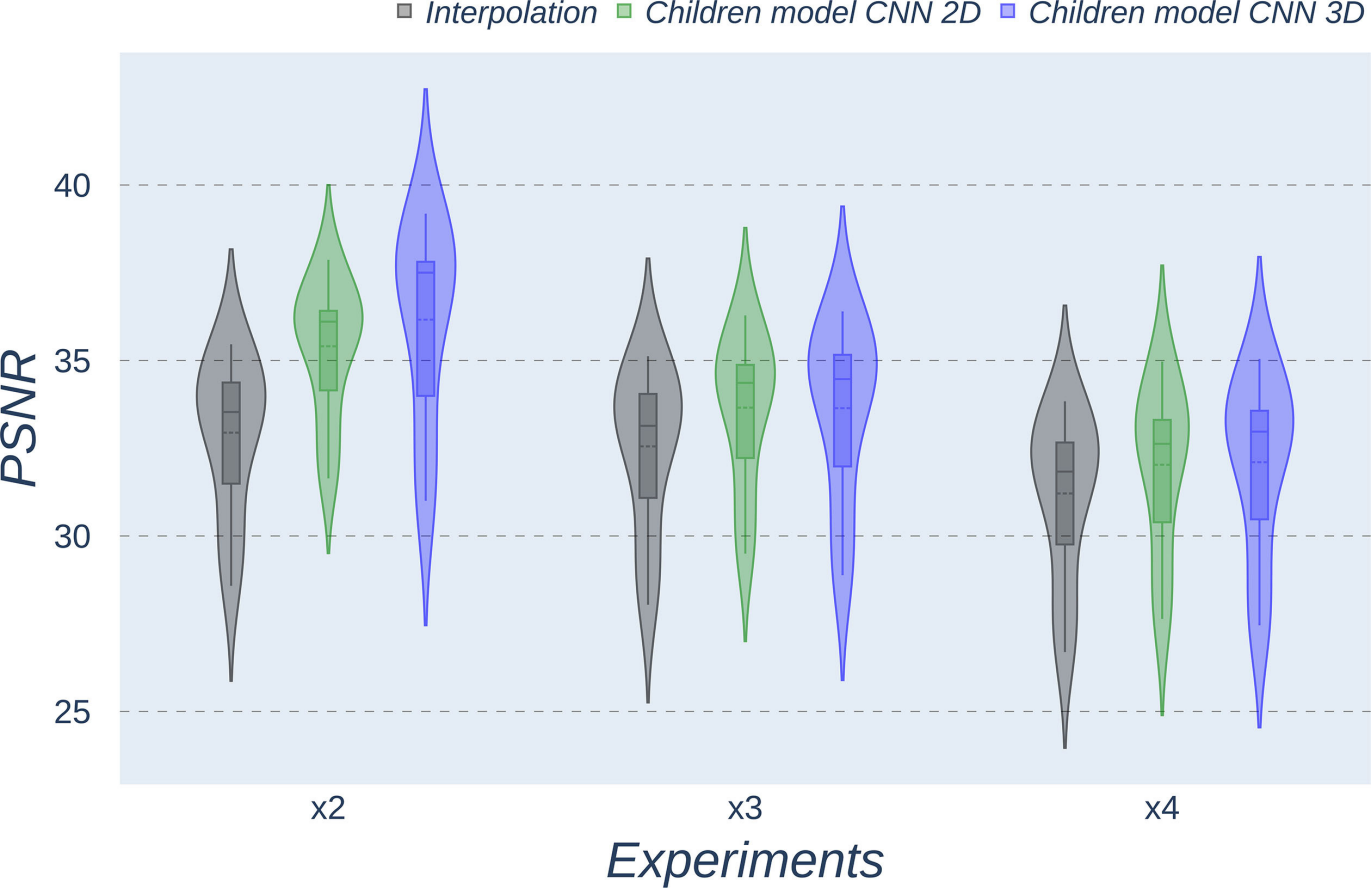
Violin plots for PSNR metric comparing the different methods at the distinct scaling factors. Each violin plot corresponds to one specific experiment, and it is calculated from dysplasic patients.

**Figure 8:**
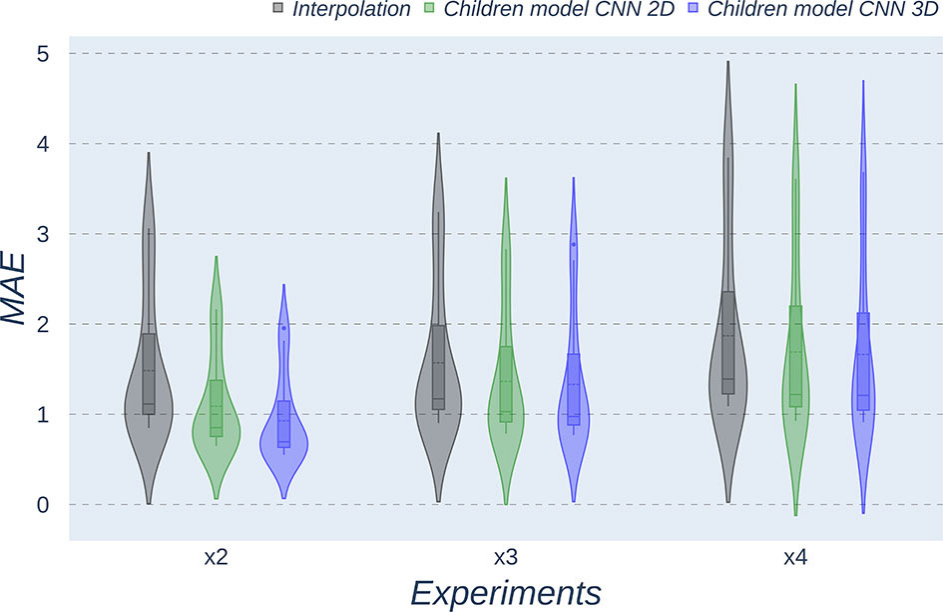
Violin plots for MAE metric comparing the different methods at the distinct scaling factors. Each violin plot corresponds to one specific experiment, and it is calculated from dysplasic patients.

**Figure 9:**
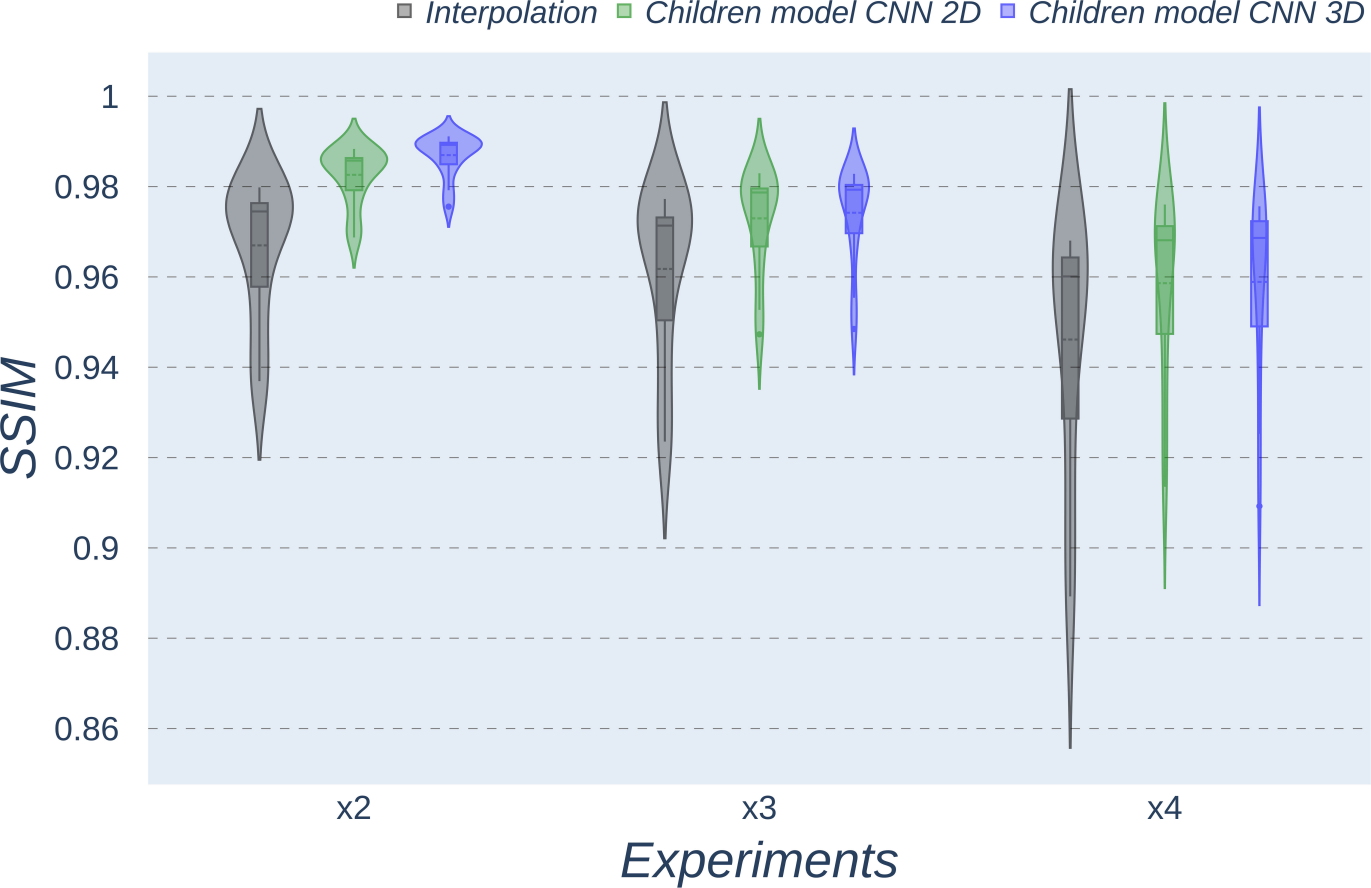
Violin plots for SSIM metric comparing the different methods at the distinct scaling factors. Each violin plot corresponds to one specific experiment, and it is calculated from dysplasic patients.

The same trends can be observed in the quantitative results over the clinical dataset, where CNNs reveal an improvement compared to interpolation methods, with the 3D CNN demonstrating the best performance in all metrics (Tables 5–7, Figures 7–9).

**Table 5:** Results for different metrics and scaling factors among dysplasic children test set using bicubic interpolation method.

**Dysplasic children**	**Bicubic interpolation**		
	**x2**	**x3**	**x4**
PSNR (dB)	32.944 ± 2.097	32.553 ± 2.188	31.211 ± 2.232
MAE	1.484 ± 0.750	1.570 ± 0.798	1.870 ± 0.966
SSIM	0.967 ± 0.002	0.962 ± 0.002	0.946 ± 0.003

**Table 6:** Results for different metrics and scaling factors among dysplasic children test set using 2D CNN method.

**Dysplasic children**	**CNN 2D**		
	**x2**	**x3**	**x4**
PSNR (dB)	35.406 ± 1.851	33.655 ± 2.088	32.028 ± 2.271
MAE	1.089 ± 0.510	1.366 ± 0.686	1.690 ± 0.903
SSIM	0.983 ± 0.006	0.9730 ± 0.011	0.9586 ± 0.020

**Table 7:** Results for different metrics and scaling factors among dysplasic children test set using 3D CNN method.

**Dysplasic children**	**CNN 3D**		
	**x2**	**x3**	**x4**
PSNR (dB)	36.164 ± 2.688	33.642 ± 2.381	32.103 ± 2.409
MAE	0.928 ± 0.457	1.332 ± 0.700	1.663 ± 0.912
SSIM	0.987 ± 0.005	0.974 ± 0.010	0.959 ± 0.020

Violin plots reveal that only the interpolation method and the adult models present outliers, confirming that CNNs are more accurate and robust methods, introducing less variability among the results for several subjects, especially when trained with specific data. PSNR shows similar trends and dispersion for different scaling factors; MAE values present a more biased widespread distribution in the values above the median than in the values below it; SSIM values are much more concentrated around the median than the rest of the metrics. Moreover, as expected, the *x2* scaling factor is the one that provides the best performance among all the scaling factors, with the 3D CNN demonstrating being the one that shows the best results.

Additionally, it is obvious that analyzing each method's results worsen as LR image size decreases. In other words, a greater scaling factor involves the loss of tissue/intensity information but implies a significant reduction on MRI acquisition time. Thus, the final acceptable scaling factor would depend on how acceptable these results with distinct scaling factors are in the final clinical practice, as shown and discussed in the following subsection.

### 3.3. Clinical Assessment

Our expert radiologist performed a clinical assessment by visually inspecting the resulting images to provide with her overall qualitative opinion of the reconstructed images regarding diagnosis (5-point Likert scale) and their ability to use them for actual diagnosis in the clinical dataset. A first inspection of the reconstructed images demonstrated that the 3D CNN method visually outperformed the 2D method; thus, subsequent analyses were performed on the resulting 3D images. It is also worth mentioning that images in the clinical dataset were acquired in two different MRI scanners implementing two different anatomical T1-weighted sequences. Thus, we have separated the corresponding analyses to assess the actual potential of the method in a clinical scenario.

Visual inspection of the images generated by our 3D CNN shows that, depending on the nature of the disease, identification of dysplasias could be straightforward or challenging depending on the baseline scaling factor. Table 8 shows the decomposition of the results for the 5-point Likert scale and the sensitivity by scaling factor and sequence. As expected, both metrics decrease their values as the scaling factor increases. The 5-point Likert scale reaches a 3.8 ± 1.0 score and a sensitivity of 0.83 ± 0.39 for the *x2* scaling factor when assessing the whole clinical dataset. Additionally, in all these cases, as expected, the CNN provides better results in those images acquired with the same type of sequence that was used for training (MPRAGE), with the 5-point Likert scale and the sensitivity increasing up to 5.0 ± 0.0 and 1.0 ± 0.0, respectively, for the same scaling factor, when analyzing the MPRAGE images only.

**Table 8:** Results for two different qualitative metrics provided by the expert radiologist.

	**Sensitivity**			**5-Point likert scale**		
	**x2**	**x3**	**x4**	**x2**	**x3**	**x4**
EFGRE3D	0.80 ± 0.42	0.70 ± 0.38	0.10 ± 0.32	3.60 ± 0.97	2.50 ± 1.08	1.60 ± 1.07
MPRAGE	1.00 ± 0.00	1.00 ± 0.00	0.75 ± 0.50	5.00 ± 0.00	4.30 ± 0.50	3.30 ± 0.50
BOTH	0.83 ± 0.39	0.75 ± 0.45	0.25 ± 0.45	3.80 ± 1.00	2.80 ± 1.30	1.80 ± 1.10

**Figure 10:**
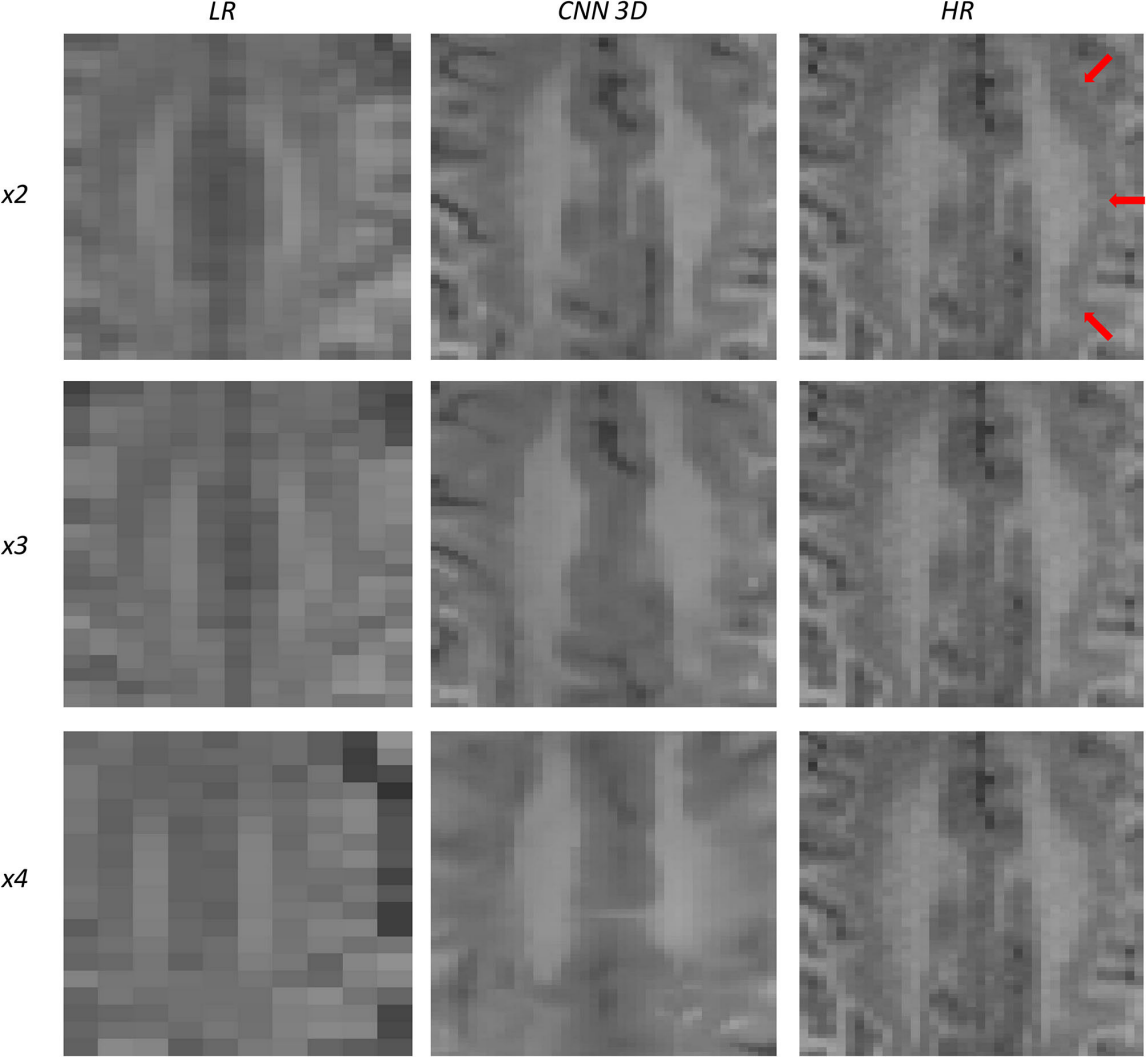
Results of both CNN methods on three scaling factors of super-resolution on images of a patient with dysplasia. A specific region of a slice is shown, with the three red arrows pointing to a lesion.

**Figure 11:**
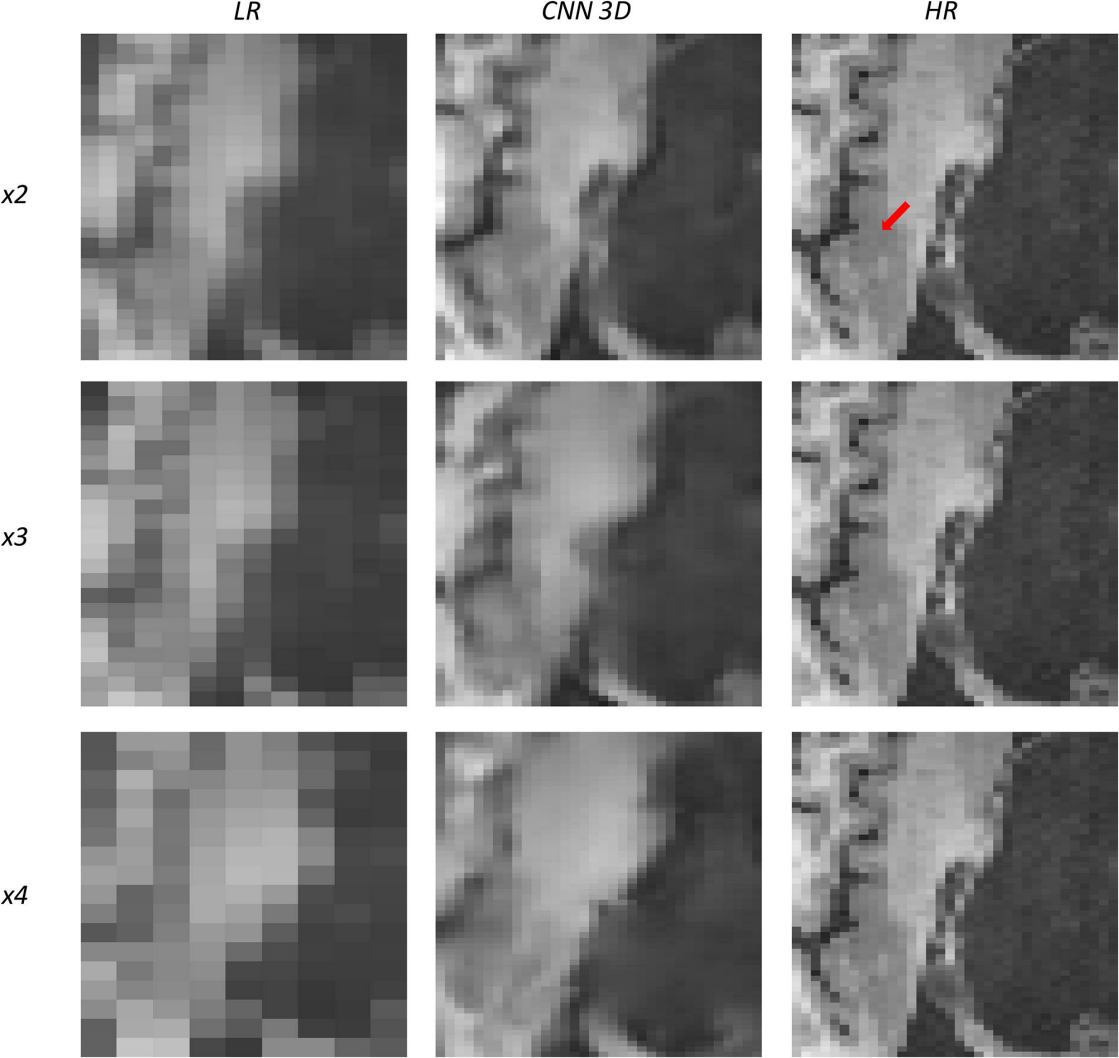
Results of both CNN methods on three scaling factors of super-resolution on images of a patient with dysplasia. A specific region of a slice is shown, with the red arrow pointing to a lesion.

**Figure 12:**
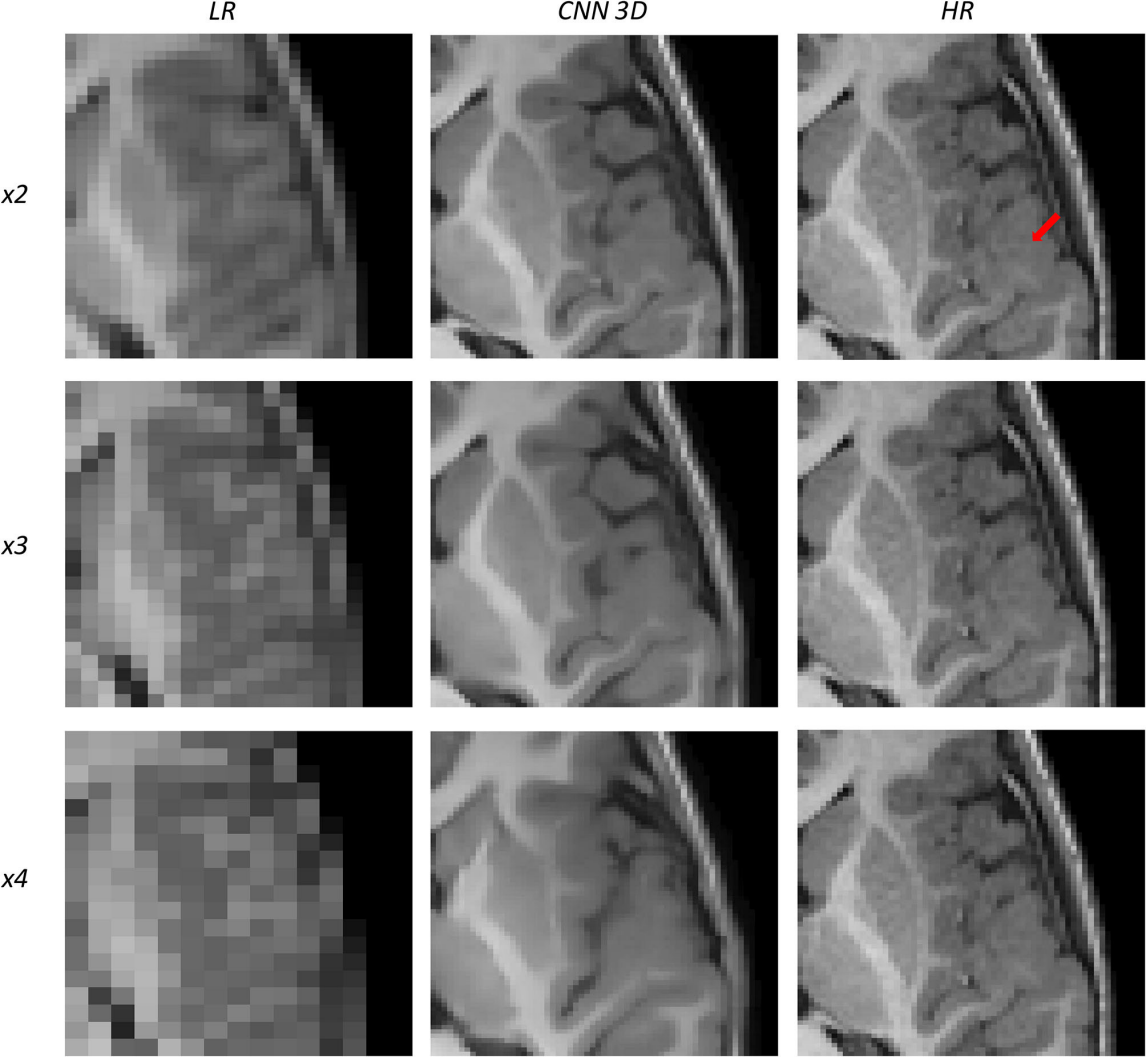
Results of both CNN methods on three scaling factors of super-resolution on images of a patient with dysplasia. A specific region of a slice is shown, with the red arrow pointing to a lesion.

**Figure 13:**
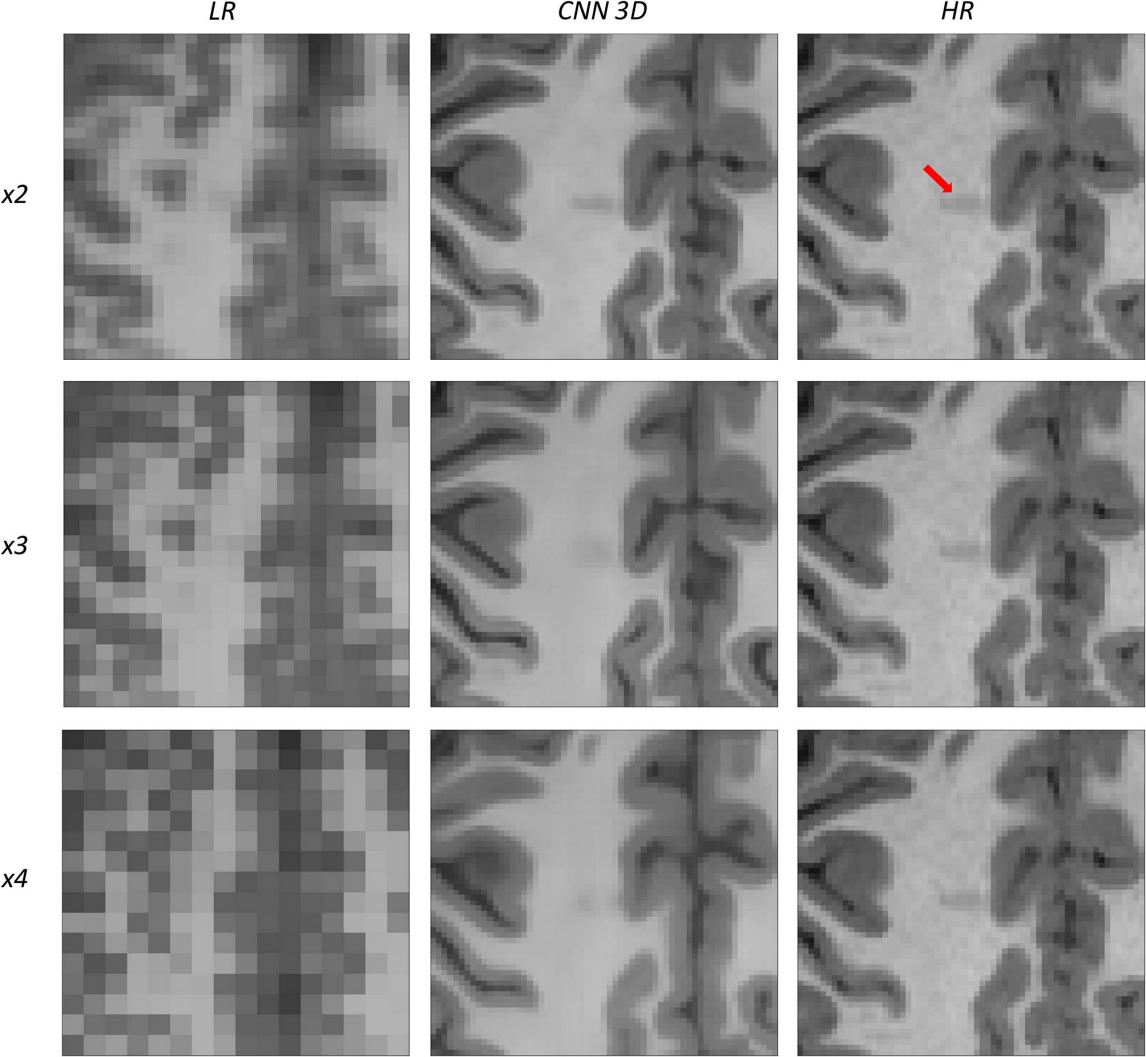
Results of both CNN methods on three scaling factors of super-resolution on images of a patient with dysplasia. A specific region of a slice is shown, with the red arrow pointing to a lesion.

Figures 10, 11 show representative examples of patients acquired using the EFGRE sequence on a GE Signa HDxt 3.0T MRI scanner, while Figures 12, 13 show representative examples of patients acquired using the MPRAGE sequence on a GE Signa Premier MRI scanner. While these images show a representative slice, our expert radiologist used the whole volume for the clinical assessment.

Figure 10 shows a 13-years-old male diagnosed with band heterotopia (double cortex syndrome), a form of diffuse gray matter heterotopia due to neuronal migration disorders. In this specific case, the CNN is not able to recover all the details for the *x4* scaling factor, producing a very blurred image; however, as we move forward to the *x3* and the *x2* scaling factors, we can observe further improvements, allowing for the assessment of the bands along the brain.

Figure 11 shows a 16-years-old male diagnosed with aqueductal stenosis, opercular syndrome, heterotopia, and a cystic brain lesion. This patient is specifically difficult to assess due to the presence of diverse diseases. Again, our method fails to recover the details for the *x4* scaling factor and improves moving forward to the *x3* and the *x2* scaling factors. The ventricle enlargement is easy to assess, but the boundaries are better displayed in the image reconstructed from the *x2* scaling factor. The opercular dysplasia can be identified in the *x3* and the *x2* scaling factors.

Figure 12 shows a 9-years-old male diagnosed with opercular syndrome and cerebral fissure malformation. As we move forward to MPRAGE images, we can see the abrupt improvement in the reconstructed images for all scaling factors. Despite the lack in detail for circumvolutions in the dysplasia for the *x4* scaling factor, the thickening of the gray matter can still be assessed. Reconstructions based on the *x3* and *x2* scaling factors allow for delineating even better dysplasia contours and edges.

Figure 13 shows a 12-years-old male diagnosed with a small cortical dysplasia. This patient is especially challenging due to the small size of the dysplasia. However, the lesion can be identified in images reconstructed from all scaling factors. The reconstruction based on the *x2* scaling factor provides an excellent contrast resolution, allowing even to identify the shape of the lesion and presenting less noise than the original HR image.

All these results show that, at least, the *x2* scaling factor is able to recover most of the details in the images, providing with good enough reconstructions to perform disease identification.

## 4. Discussion

Brain related pathologies often need MRI as a decisive imaging acquisition technique to find the correct diagnosis. Nevertheless, motion artifacts usually appear in pediatric MRI and a method is required to improve the resolution from LR images correctly acquired in a reduced examination time to exclude the use of either sedatives or anesthetics. This project has been based on the design and implementation of a CNN that learns how to automatically increase the resolution of an LR MRI without the need to know its HR counterpart.

In this work, we have proved how the use of CNNs can satisfactorily solve the initial SR proposal. The database chosen consists of a sufficient number of subjects for the CNN to be able to generalize. In addition, the preprocessing carried out on the original MRI images has managed to adapt them correctly as input to the CNN and the drawback of the artificial generation of the LR database has not adversely affected the performance of the CNN. We obtain visual-pleasing results which agree with the metrics employed to assess the correlation and difference between each pair of images. No matter which scaling factor is utilized, different brain tissues can always be differentiated and most of their details are maintained on the SR image.

We have also carried out an MRI simulation for the original matrix size with 1 mm isotropic voxel and its related scaling factor images (2, 3, and 4 mm). The decrease in acquisition time was around 50% for the first scaling factor and 60% for the second one. This reduction could feed physicians with good quality MRI that aid the clinical routine. Depending on the MRI scanner, the time employed to complete a study ranges from 30 min to 2 h and brain MRI takes an average of 45 min or even longer (Slovis, 2011). It then becomes possible to decrease the examination time by about 20 min.

Our results are compared with the state of art methods that use different datasets than ours. These differences include the MRI sequence, the size and age range of the database, and the scanner specifications. We, therefore, need to be careful when comparisons are made with reviewed literature. Furthermore, the LR images were not directly acquired from the scanner and we made the assumption of resemblance between our artificial LR images and the hypothetical LR acquired ones, in order to be able to discuss the different results.

In future work, it would be desirable to extrapolate the results obtained to different datasets and other modalities of MRI, such as T2-weighted, Diffusion Tensor imaging, or functional MRI. Transfer learning from this work model could be applied to those other modalities and build a stronger method that does not depend on a specific MRI modality. If possible, LR images should be acquired directly from MRI equipment and physicians should check in a clinical study that SR images preserve the same diagnostic value than original acquired images.

## 5. Conclusion

This paper proposes a new CNN pipeline to perform superresolution on pediatric MRI and allows a reduction of MRI examination time. We assessed several CNN architectures on different scaling factors. Our work represents a substantial innovation in the pediatric MRI field, proposing an initial starting point to eliminate the need for a sedation protocol among the infant population.

## Data Availability Statement

The healthy participants dataset analyzed in this study is publicly available in the Open Neuro Repository (https://openneuro.org/datasets/ds000228/versions/1.1.0). The clinical image dataset is not publicly available due to IRB restrictions. Further inquiries can be directed to the corresponding author.

## Ethics Statement

Dysplasic children MRI data were retrospectively collected and anonymized from existing clinical datasets after review and approval of the Research Committee from Hospital Universitario Quirónsalud (Madrid, Spain). Patient consent was not required for the study on human participants in accordance with local legislation and institutional requirements.

## Author Contributions

JM-M and AG-B developed and implemented the methods. JM-M was responsible for data curation, conduction of the experiments, and writing the first draft. MJ was responsible for clinical data curation, conduction of the clinical assessments, and validation of the results concerning the pathological dataset. JM-M, AG-B, MJ, NM, and AT-C analyzed and interpreted the final data and results. MJ, NM, and AT-C critically revised the manuscript. AT-C was responsible for conceptualization, methodology, supervision, resources, and overall project administration. All authors listed have made a substantial, direct and intellectual contribution to the work and design of the study, contributed to manuscript revision, proofreading, and approved the submitted version for publication.

## Conflict of Interest

The authors declare that the research was conducted in the absence of any commercial or financial relationships that could be construed as a potential conflict of interest.

## Publisher's Note

All claims expressed in this article are solely those of the authors and do not necessarily represent those of their affiliated organizations, or those of the publisher, the editors and the reviewers. Any product that may be evaluated in this article, or claim that may be made by its manufacturer, is not guaranteed or endorsed by the publisher.

## References

[B1] Ahmad R. Hu H. H. Krishnamurthy R. Krishnamurthy R. (2018). Reducing sedation for pediatric body MRI using accelerated and abbreviated imaging protocols. Pediatr. Radiol. 48, 37–49. 10.1007/s00247-017-3987-6 29292482

[B2] Chaudhari A. S. Fang Z. Kogan F. Wood J. Stevens K. J. Gibbons E. K.. (2018). Super-resolution musculoskeletal MRI using deep learning. Magn. Reson. Med. 80, 2139–2154. 10.1002/mrm.27178 29582464 PMC6107420

[B3] Chen Y. Xie Y. Zhou Z. Shi F. Christodoulou A. G. Li D. (2018). “Brain MRI super resolution using 3D deep densely connected neural networks,” in 2018 IEEE 15th International Symposium on Biomedical Imaging (ISBI 2018) (Washington, DC: IEEE), 739–742.

[B4] Du J. He Z. Wang L. Gholipour A. Zhou Z. Chen D.. (2020). Super-resolution reconstruction of single anisotropic 3D MR images using residual convolutional neural network. Neurocomputing 392, 209–220. 10.1016/j.neucom.2018.10.102

[B5] Edwards A. D. Arthurs O. J. (2011). Paediatric MRI under sedation: is it necessary? what is the evidence for the alternatives? Pediatric radiology 41, 1353–1364. 10.1007/s00247-011-2147-7 21678113

[B6] Gholipour A. Estroff J. A. Warfield S. K. (2010). Robust super-resolution volume reconstruction from slice acquisitions: application to fetal brain MRI. IEEE Trans. Med. Imaging 29, 1739–1758. 10.1109/TMI.2010.2051680 20529730 PMC3694441

[B7] Harned Ii R. K. Strain J. D. (2001). MRI-compatible audio/visual system: impact on pediatric sedation. Pediatr. Radiol. 31, 247–250. 10.1007/s002470100426 11321741

[B8] Khan J. J. Donnelly L. F. Koch B. L. Curtwright L. A.. (2007). A program to decrease the need for pediatric sedation for CT and MRI. Appl. Radiol. 36, 30. 10.37549/AR1505

[B9] Lustig M. Donoho D. Pauly J. M. (2007). Sparse mri: The application of compressed sensing for rapid mr imaging. Magn. Reson. Med. 58, 1182–1195. 10.1002/mrm.21391 17969013

[B10] Lyu Q. You C. Shan H. Wang G. (2018). Super-resolution MRI through deep learning. arXiv [Preprint] arXiv:1810.06776. 10.1117/12.2530592

[B11] McGee K. (2003). The role of a child life specialist in a pediatric radiology department. Pediatr. Radiol. 33, 467–474. 10.1007/s00247-003-0900-2 12819835

[B12] Peled S. Yeshurun Y. (2001). Superresolution in MRI: application to human white matter fiber tract visualization by diffusion tensor imaging. Magn. Reson. Med. 45, 29–35. 10.1002/1522-2594(200101)45:1andlt;29::AID-MRM1005andgt;3.0.CO;2-Z 11146482

[B13] Pham C.-H. Ducournau A. Fablet R. Rousseau F. (2017). “Brain MRI super-resolution using deep 3D convolutional networks,” in 2017 IEEE 14th International Symposium on Biomedical Imaging (ISBI 2017) (Melbourne, VIC: IEEE), 197–200.

[B14] Pham C.-H. Tor-Díez C. Meunier H. Bednarek N. Fablet R. Passat N.. (2019). Multiscale brain MRI super-resolution using deep 3D convolutional networks. Comput. Med. Imaging Graph. 77, 101647. 10.1016/j.compmedimag.2019.101647 31493703

[B15] Qiu D. Zhang S. Liu Y. Zhu J. Zheng L. (2020). Super-resolution reconstruction of knee magnetic resonance imaging based on deep learning. Comput. Methods Progr. Biomed. 187, 105059. 10.1016/j.cmpb.2019.105059 31582263

[B16] Richardson H. Lisandrelli G. Riobueno-Naylor A. Saxe R. (2018). Development of the social brain from age three to twelve years. Nat. Commun. 9, 1–12. 10.1038/s41467-018-03399-2 29531321 PMC5847587

[B17] Ronneberger O. Fischer P. Brox T. (2015). U-net: “Convolutional networks for biomedical image segmentation,” in *International Conference on Medical Image Computing and Computer-Assisted Intervention* (Munich: Springer), 234–241.

[B18] Rueda A. Malpica N. Romero E. (2013). Single-image super-resolution of brain MR images using overcomplete dictionaries. Med. Image Anal. 17, 113–132. 10.1016/j.media.2012.09.003 23102924

[B19] Sánchez I. Vilaplana V. (2018). Brain MRI super-resolution using 3D generative adversarial networks. arXiv [Preprint] arXiv:1812.11440. 10.48550/arXiv.1812.11440

[B20] Schulte-Uentrop L. Goepfert M. S. (2010). Anaesthesia or sedation for MRI in children. Curr. Opin. Anesthesiol. 23, 513–517. 10.1097/ACO.0b013e32833bb524 20531170

[B21] Shi F. Cheng J. Wang L. Yap P.-T. Shen D. (2015). LRTV: MR image super-resolution with low-rank and total variation regularizations. IEEE Trans. Med. Imaging 34, 2459–2466. 10.1109/TMI.2015.2437894 26641727 PMC5572670

[B22] Slovis T. L. (2011). Sedation and anesthesia issues in pediatric imaging. Pediatr. Radiol. 41, 514–516. 10.1007/s00247-011-2115-2 21847732

[B23] Smith C. Bhanot A. Norman E. Mullett J. Bilbo S. McDougle C.. (2019). A protocol for sedation free MRI and PET imaging in adults with autism spectrum disorder. J. Autism. Dev. Disord. 49, 3036–3044. 10.1007/s10803-019-04010-3 31004246

[B24] Tan C. Zhu J. Lio P. (2020). “Arbitrary scale super-resolution for brain MRI images,” in IFIP International Conference on Artificial Intelligence Applications and Innovations (Neos Marmaras: Springer), 165–176.

[B25] Verriotis M. Moayedi M. Sorger C. Peters J. Seunarine K. Clark C. A.. (2020). The feasibility and acceptability of research magnetic resonance imaging in adolescents with moderate-severe neuropathic pain. Pain Rep. 5, e807. 10.1097/PR9.0000000000000807 32072101 PMC7004507

[B26] Wang J. Chen Y. Wu Y. Shi J. Gee J. (2020). “Enhanced generative adversarial network for 3d brain mri super-resolution,” in Proceedings of the IEEE/CVF Winter Conference on Applications of Computer Vision (Snowmass, CO: IEEE), 3627–3636.

[B27] Yan Z. Lu Y. (2009). “Super resolution of MRI using improved IBP,” in 2009 International Conference on Computational Intelligence and Security, Vol. 1 (Beijing: IEEE), 643–647.

[B28] You S. Liu Y. Lei B. Wang S. (2020). Fine perceptive gans for brain MR image super-resolution in wavelet domain. arXiv [Preprint] arXiv:2011.04145. 10.48550/arXiv.2011.04145 35254996

[B29] Zeng K. Zheng H. Cai C. Yang Y. Zhang K. Chen Z. (2018). Simultaneous single-and multi-contrast super-resolution for brain MRI images based on a convolutional neural network. Comput. Biol. Med. 99, 133–141. 10.1016/j.compbiomed.2018.06.010 29929052

[B30] Zeng W. Peng J. Wang S. Liu Q. (2020). A comparative study of cnn-based super-resolution methods in mri reconstruction and its beyond. Signal Process. 81, 115701. 10.1016/j.image.2019.115701

[B31] Zhang Y. Yap P.-T. Chen G. Lin W. Wang L. Shen D. (2019). Super-resolution reconstruction of neonatal brain magnetic resonance images via residual structured sparse representation. Med. Image Anal. 55, 76–87. 10.1016/j.media.2019.04.010 31029865 PMC7136034

